# Intracellular galectin-7 expression in cancer cells results from an autocrine transcriptional mechanism and endocytosis of extracellular galectin-7

**DOI:** 10.1371/journal.pone.0187194

**Published:** 2017-11-08

**Authors:** Nathalie Bibens-Laulan, Yves St-Pierre

**Affiliations:** INRS-Institut Armand-Frappier, 531 Boul. des Prairies, Laval, QC, Canada; University of British Columbia, CANADA

## Abstract

The β-galactoside binding protein galectin-7 (gal-7) is constitutively expressed at abnormally high levels in the outside milieu and intracellular compartments of many types of epithelial cancer cells, most notably in aggressive forms of ovarian and breast cancer. It is thus of utmost importance to understand how gal-7 traffics between both intracellular and extracellular compartments to develop novel drugs that target the protumorigenic functions of galectin-7. In the present work, we report that extracellular gal-7 plays a central role in controlling intracellular gal-7 in cells. It does so via two distinct yet complementary mechanisms: firstly by increasing the transcriptional activation of *lgals7* gene transcription, and secondly via re-entry into the cells. Increased mRNA levels were dose- and time-dependent and occur in all cell lines tested, including ovarian and breast cancer cell lines. Addition of recombinant gal-7 to MDA-MB-231 transfected with a luciferase reporter vector containing response elements of the *lgals7* promoter indicated that increased mRNA level of *lgals7* occurs via *de novo* gene transcription. Re-entry of extracellular gal-7 inside cells was rapid, and reached cytosolic and mitochondrial compartments. Taken together, these findings reveal the existence of a positive self-amplification pathway that regulates intracellular gal-7 expression in breast and ovarian cancer cells.

## Introduction

Galectins are intracellular small molecular weight soluble proteins that are released in the extracellular space via a non-classical export mechanism. Once in the extracellular space, they bind to repeating units of high density O-glycans on the peptide backbone of membrane receptors, facilitating the packing of glycosylated receptors into an ordered cross-linked lattice at the cell surface [1–3]. In the cytosol, they accomplish various cellular functions by interacting with multiple ligands using CRD- and CRD-independent interactions [4]. This is particularly true for galectin-7 (gal-7). We and others have found that gal-7 is constitutively expressed in the cytosol of multiple types of cancer cells of epithelial origin, most notably breast and ovarian cancer cells [5–7]. The mechanisms responsible for such constitutive expression of gal-7 intracellularly, however, remain largely unknown although it is logical to assume that gal-7 in cancer cells is regulated, at least in part, at the transcriptional level via DNA methylation and the implication of specific transcription factors, such as mutant p53, Nf-kB, and the CCAAT/enhancer-binding protein beta-2 isoform (CEBPβ-2) [8–10]. Because galectins are well known for their ability penetrate cells via endocytosis following binding to cell surface glycoreceptors [11,12], another possibility is that cytosolic gal-7 originates from endocytic uptake from the pool present in the extracellular milieu. Solving this issue is critical for the design of gal-7-specific drugs aimed at inhibiting gal-7 protumorigenic functions, most notably in high fatality cancer for which no effective treatment exists. In the present work, we provide evidence that expression of gal-7 inside cancer cells results from both an autocrine transcriptional mechanism and the endocytosis of extracellular gal-7.

## Material and methods

### Reagents and cell lines

The breast MDA-MB-231, MCF-7, MDA-MB-468 cell lines and the human fibrosarcoma HT1080 cell lines were obtained from the American Tissue Culture Collection (ATCC, Manassas, VA). The ovarian cancer cell lines were kindly provided by Dr. E. Asselin (University of Quebec in Trois-Rivières). The ovarian human A2780 and OVCAR-3 cell lines were maintained in RPMI 1640 medium and SKOV-3 cell line was maintained in McCoy’s 5A medium supplemented with 2 mM L-glutamine, 4-(2-hydroxyethyl)-1-piperazine ethane sulfonic acid (HEPES) buffer and 15% (vol/vol) FBS. The other cell lines were maintained in culture in Dulbecco modified Eagle Medium (DMEM) supplemented with 2 mM L-glutamine, 1mM sodium pyruvate 10 mM, HEPES, and 10% (vol/vol) FBS. All cell culture products were purchased from Life Technologies (Burlington, ON, Canada). Pitstop-2 was obtained from Abcam (Toronto, ON, Canada) and Dynasore from Sigma-Aldrich (Oakville, ON, Canada). Anti-human galectin-7 was purchased from R&D Systems (Minneapolis, MN, USA) while anti-lamin A/C, anti-Cox IV and anti-β-tubulin were obtained from Cell Signaling (Danvers, MA, USA). All other chemicals, including anti-β-actin and anti-FITC antibodies, were from Sigma Aldrich (St. Louis, MO, USA) unless stated otherwise.

### RNA extraction and semi-quantitative RT-PCR

Total RNA was isolated from cells using Trizol reagent according to the manufacturer’s instructions (Invitrogen, Burlington, ON, Canada). After reverse transcription with an Omniscript reverse transcriptase kit from QIAGEN (Toronto, ON, Canada), cDNA was amplified using the following conditions: 94°C for 1 min, followed by 30–40 cycles of the following: 94°C for 1 min, 58–64°C for 1min (depending on the primers) for hybridization temperature, and 72°C for 1 min, followed by a final extension step at 72°C for 10 min. PCR assays using equal amounts of RNAs that were reverse-transcribed and amplified by PCR with genes specific primers (**Table 1**) confirmed that the amplification was in the linear range for each gene. As an internal control, amplification of glyceraldehyde-3-phosphate dehydrogenase (GAPDH) mRNA. PCR reactions were performed in a thermal cycler (MJ Research, Watertown, MA) and amplicons analyzed by electrophoresis on agarose gels using SYBR Safe DNA staining (Invitrogen) and UV illumination.

**Table 1:** 

**Gene**	**Sense**	**Antisense**
MMP-9	5’-CAA CAT CAC CTA TTG GAT CC-3’	5’-CGG GTG CAC CTA TTG GAT CC-3’
GAPDH	5’-CGG AGT CAA CGG ATT TGG TCG TAT-3’	5’-CAG AAG TGG TGG TAC CTC TTC CGA-3’
*Lgals3*	5’-ATG GCA GAC AAT TTT TCG CTC C-3’	5’-ATG TCA CCA GAA ATT CCC AGT T-3’
*Lgals7*	5’-ATG TCC AAC GTC CCC CAC AAG-3’	5’-TGA CGC GAT GAT GAG CAC CTC-3’

### Vectors, transfection and luciferase assays

The pGL3 Basic luciferase reporter vector encoding for the human *lgals7* promoter region has been described [8]. For transfection, cells were plated at equal density 16–24 h before transfection. Cells were then transfected using DNAfectin 2100 (ABM, Richmond, BC, Canada) according to the manufacturer’s protocol. After transfection, cells were incubated in complete medium at 37°C in 5% CO_2_ for the indicated periods of time and subjected to a dual reporter assay. Luciferase activity was measured using the Luciferase Assay System protocol (Promega, Madison, WI, USA) and a luminometer (Lumat LB 9507, Berthold). β-galactosidase activity was measured using a colorimetric enzyme assay using the Luminescent β-Galactosidase Detection Kit II according to the manufacturer’s instructions (Clontech Laboratories, Mountain View, CA). Luciferase expression levels were normalized to the levels of β-galactosidase expression.

### Western blot analysis

Whole cell extracts were homogenized in RIPA lysis buffer (Thermo Fisher Scientific, Rockford, IL) containing protease inhibitors (Roche-Diagnostic, Mississauga, ON, Canada) following the manufacturer’s instructions. Mitochondria, nuclear and cytoplasm proteins were extracted using commercial kits (Sigma-Aldrich). Extracellular proteins were collected from cell supernatant and concentrated under vaccum. Equal amounts of proteins were loaded and separated on a 15% SDS-PAGE gel. After transfer, nitrocellulose membranes were first blocked with in a 5% milk in PBS/0.05% Tween 20 solution for 1h and subsequently blotted overnight at 4°C with the primary antibody. Secondary antibodies consisted of horseradish peroxidase (HRP)-conjugated anti-rabbit, anti-mouse, or anti-goat antibodies (GE Healthcare, Mississauga, ON, Canada). Detection was performed using the enhanced chemiluminescence (ECL) method.

### Production of recombinant galectin

Production of human recombinant gal-7 was carried out as described using a pET-22b(+) plasmid encoding a synthetic, codon-optimized cDNA [13]. In some experiments, recombinant gal-7 was labeled with fluorescein isothiocyanate (FITC) as described [13]. FITC-labeled gal-7 was purified using a PD-10 Sepharose column (GE healthcare) and eluted with PBS.

### Confocal analysis

Cells were fixed in paraformaldehyde 3% [w/v] for 15 min, permeabilized in PBS/Triton X-100 0.1% [v/v] for 5 min and blocked 30 min at 4°C in 1% [v/v] BSA diluted in PBS (PBA) before addition of the antibodies. All antibodies were diluted in PBA and all washing steps were performed with PBS. Nuclei were stained with ProLong Gold Antifade Reagent with DAPI (Life Technologies). For live cell imaging, cells were seeded in 6-well plates and incubated with FITC-labeled recombinant gal-7 for time-courses ranging from 5 min to 1 hr. DAPI and WGA were used as counterstain. 3D confocal time-lapse imaging was performed at 37°C in a humidified, temperature- and CO_2_-controlled live cell chamber. Images were collected sequentially in three channels (633, 488 and 561nm laser) every 15 sec. Confocal image z-stacks of live cells were recorded with a frame size of 724x724 pixels, a pixel size of 70 nm (0.07μm x 0.07 μm x 1.00 μm) and a z-step size of 1.00 μm every 1.75 seconds. Images shown are maximum intensity projections of few mid-z-sections. All immunofluorescence analyses were carried out using a LSM 780 laser-scanning microscope (Zeiss, Jena, Germany).

### Statistical analysis

Statistical significance was calculated using the unpaired Student’s t-test. Results were considered statistically significant at *p* ≤ 0.05.

## Results

### Autocrine regulation of *lgals7* in cancer cells

**Fig 1:**
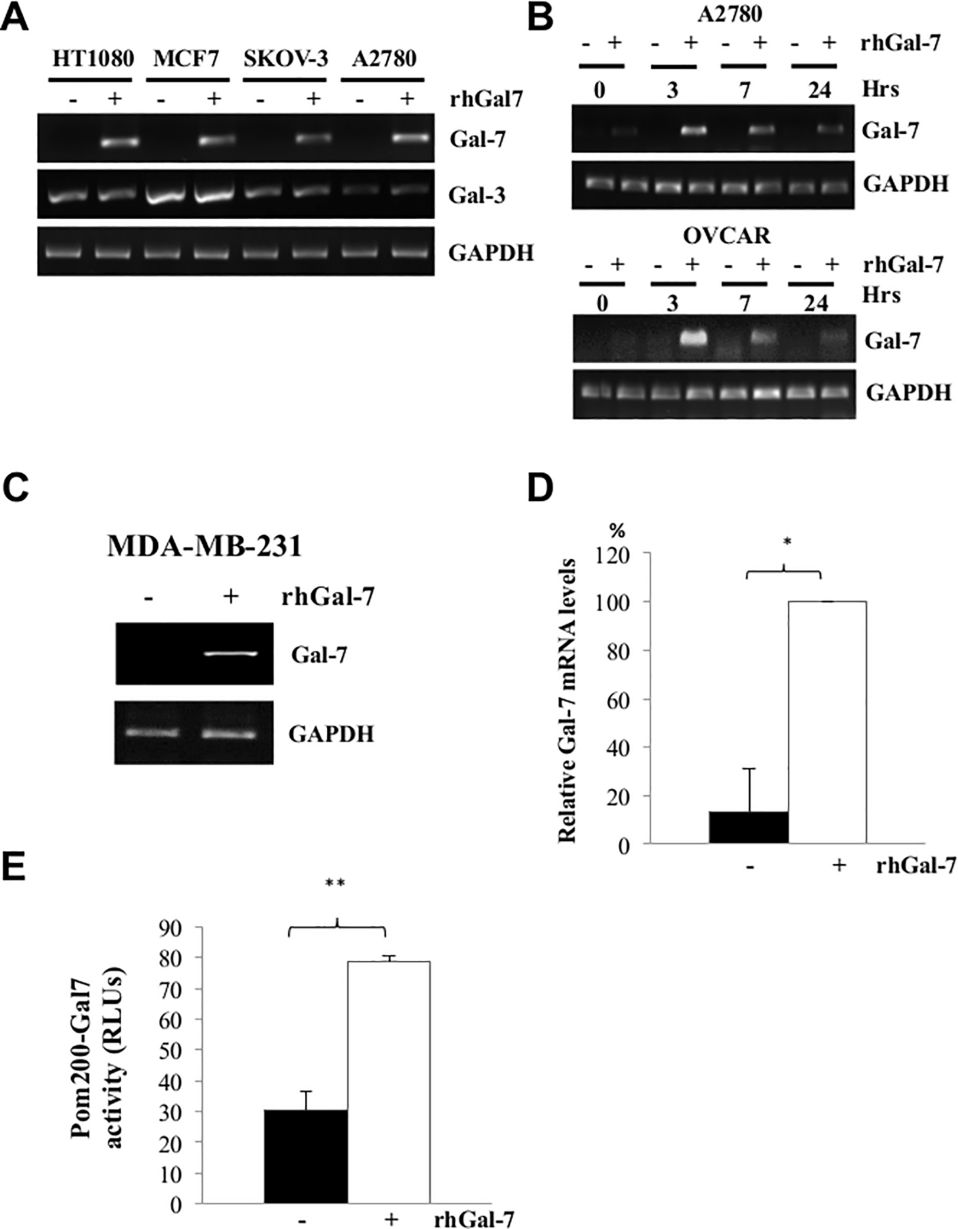
Increase mRNA levels of human gal-7 in cancer cells following stimulation with human recombinant galectin-7 (rhGal-7). (**A**) Levels of transcripts were measured by RT-PCR 16 h after addition of rhGal-7. (**B**) Kinetic analysis of *lgals7* mRNA expression induced by rhGal-7 (5μM) in OVCAR and A2780 cells. (**C**) mRNA level of *lgals7* in MDA-MB-231 cells after treatment with rhGal-7 (5μM). (**D**) Quantitative analyses of *lgals7* mRNA levels in MDA-MB-231 as measured by imaging densitometry. (* *p* ≤ 0.05). (**E**) Luciferase activity measured in protein extracts collected from MDA-MB-231 cells transfected with a luciferase reporter vector containing *p200-gal7* promoter following treatment with rhGal-7 (** *p* ≤ 0.005). (**F**) *lgals7* expression induced by rhGal-7 at different concentrations. Data are representative of at least three independent experiments.

Sustained expression of gal-7 is commonly observed inside and outside aggressive carcinoma cells. Because binding of extracellular galectins to cell surface receptors is well known to trigger gene expression in a wide spectrum of cell types, we first investigated whether extracellular gal-7 can induce its own expression via an autocrine regulatory loop. For this purpose, cancer cells were incubated with increasing concentrations of recombinant human gal-7 for different period of times. Cells were then harvested and *lgals7* gene expression measured by standard RT-PCR. Our results showed that exposure of cancer cells to gal-7 did induce *lgals7* mRNA expression (**Fig 1A**). Gal-7 did not induce or modulate expression of *lgals3*, indicating that this autocrine regulation was specific. This induction of *lgals7* by recombinant gal-7 was rapid and could readily be detected 3 hr. after addition of gal-7 (**Fig 1B**). Upregulation of *lgals7* by recombinant gal-7 was observed in both ovarian and breast cancer cell lines, including the human breast cancer cell line, MDA-MB-231 (**Fig 1C and 1D**). Experiments where addition of recombinant gal-7 to MDA-MB-231 transfected with a luciferase reporter vector containing response elements of the *lgals7* promoter indicated that increased mRNA level of gal-7 in cancer cells occurs via *de novo* gene transcription (**Fig 1E**). Similar results were obtained using MCF-7 and HT1080 cells (**S1 Fig**). Upregulation of *lgals7* by recombinant gal-7 was found to be dose-dependent (**Fig 1F**). Treatment with cycloheximide (CHX) partically inhibited the pool of intracellular gal-7 in MDA-MB-231 cells, consistent with *de novo* expression of gal-7 at the protein level (**S2 Fig)**. Taken together, these findings reveal the existence of a gal-7 autocrine positive feedback in ovarian and breast cancer cells.

### Increased intracellular expression of gal-7

**Fig 2:**
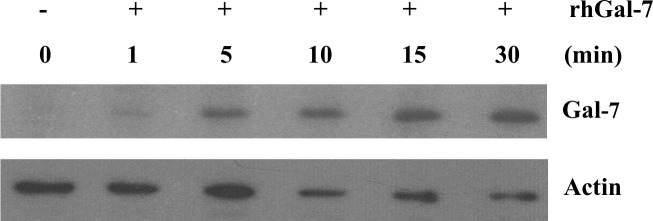
Induction of galectin-7 in MDA-MB-231 cells is CRD-dependent. Expression of galectin-7 was measured by Western blot at different times following stimulation with rhGal-7.

**Fig 3:**
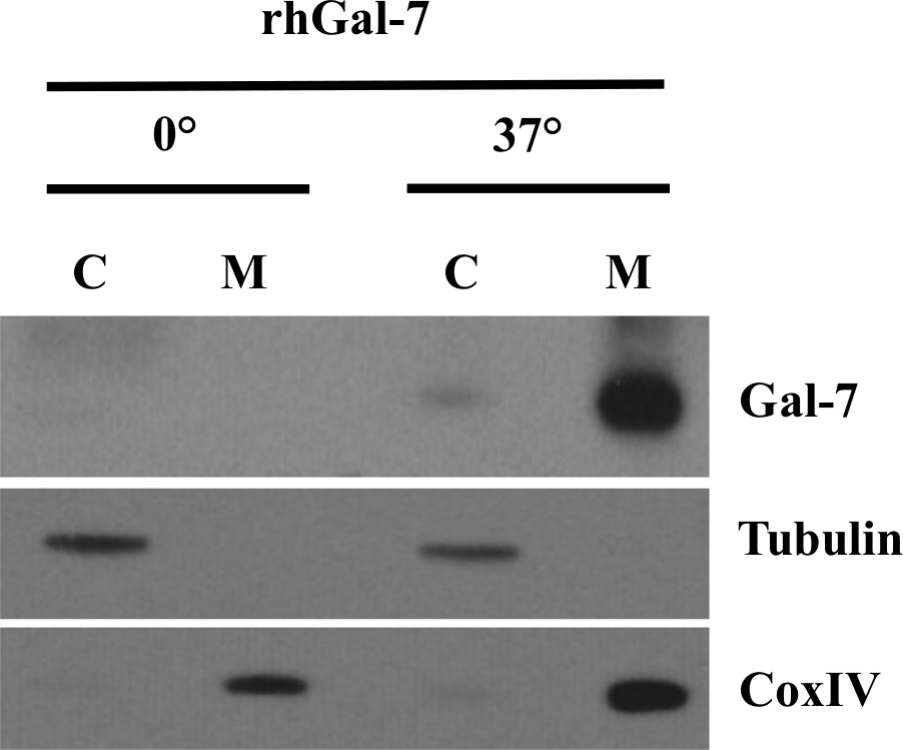
Mitochondrial localization of galectin-7. Western blot analysis in MDA-MB-231 cells showing expression of cytosolic and mitochondrial galectin-7 15 min after stimulation with rhGal-7 at 0°C or 37°C. Tubulin and CoxIV were used as controls for cytosolic and mitochondrial extracts respectively.

Western blot analysis of cell lysates collected from MDA-MB-231 cells following treatment with recombinant human gal-7 showed a time-dependent increase of gal-7 at the protein level that was detectable at 1–5 min post-incubation (**Fig 2**). This induction was temperature dependent (**Fig 3**). Increased expression of gal-7 inside the cells occurred in both cytosolic and mitochondrial compartments where gal-7 is normally found (**Fig 3**).

### Endocytosis of extracellular gal-7

**Fig 4:**
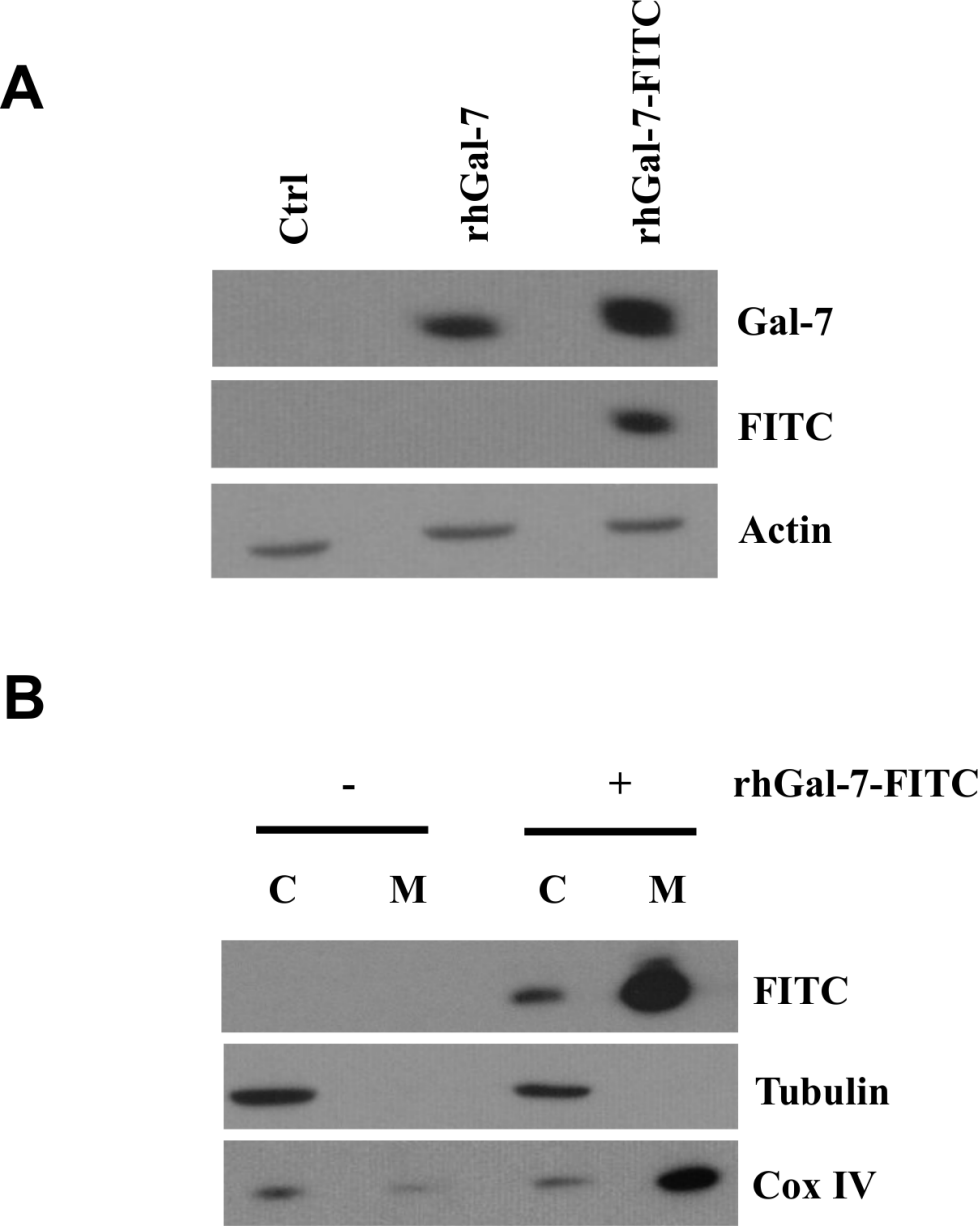
Western blot analysis showing endocytosis of FITC-labeled galectin-7. (**A**) Western blot analysis of cell lysates collected from MDA-MB-231 cells treated with rh-Gal-7 or FITC-rhGal-7 (15 min. post-treatment) (**B**) Expression of cytosolic or mitochondrial galectin-7 following treatment with rhGal-7 or FITC-rhGal-7 (15 min. post-treatment).

**Fig 5:**
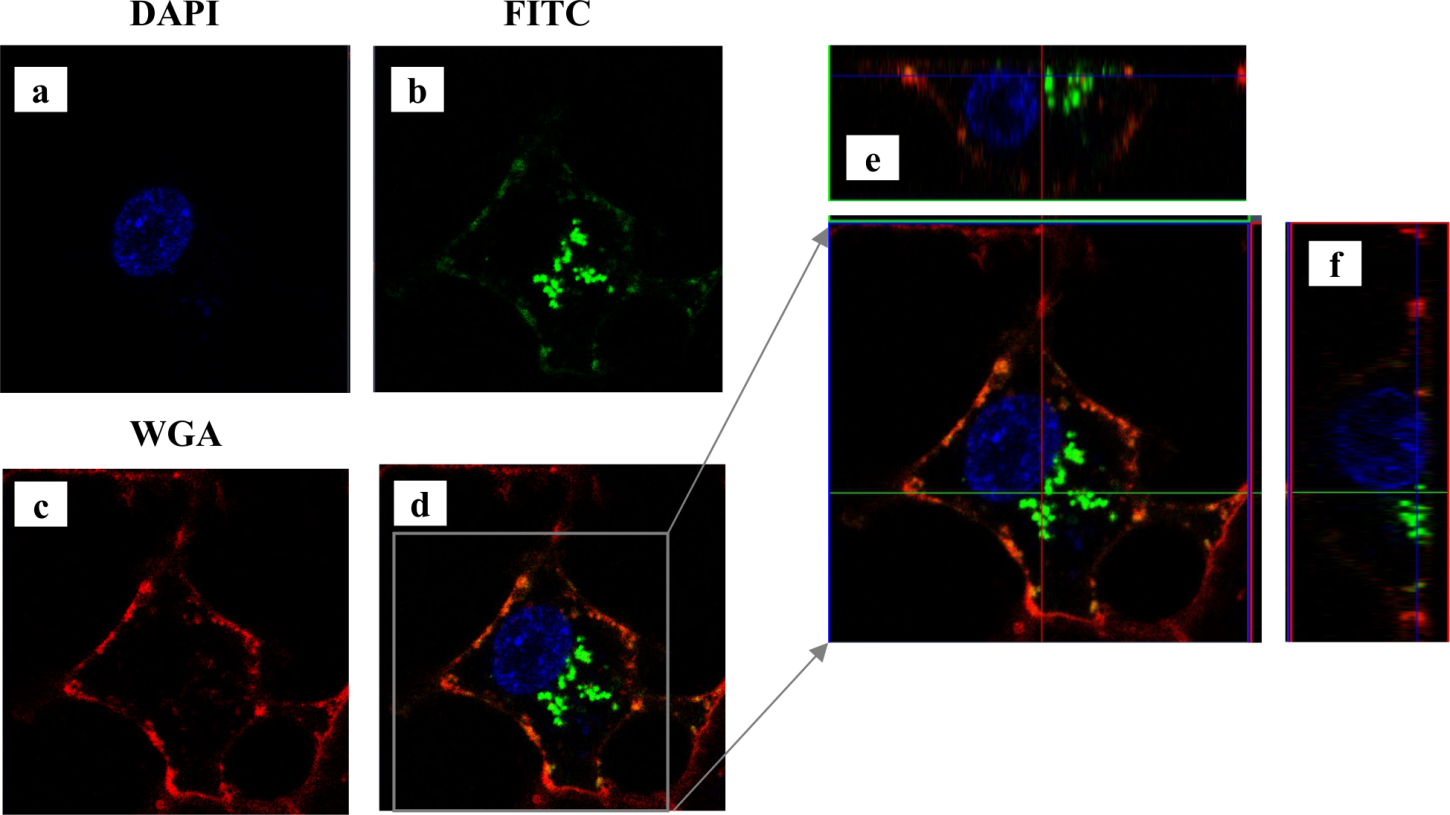
Endocytosis FITC-labeled galectin-7 viewed by confocal microscopy. Confocal microscopy of MDA-MB-231 cells treated with FITC-rhGal-7 during 25 minutes. Staining with DAPI (a) and WGA (c), which target the nucleus and plasma membrane respectively, are shown. In (b), staining of FITC-gal-7. (e) and (f) show cross sections of the cell.

**Fig 6:**
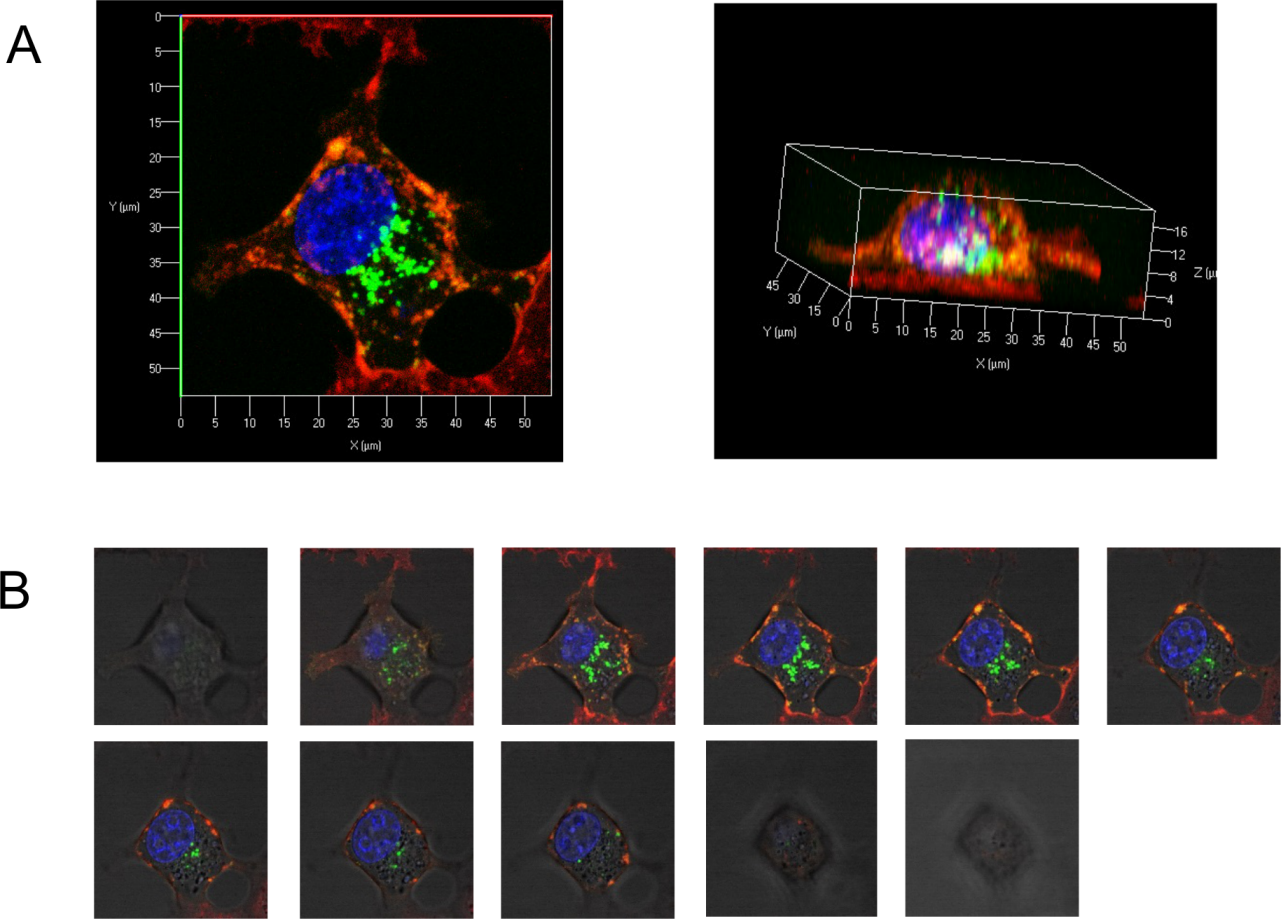
Confocal microscopy of 3D sections of MDA-MB-231 cells treated with FITC-rhGal-7. (**A**) 3D reconstruction of endocytosed FITC-rhGal-7 as described in Fig 5. In (**B**), multiple cross sections in the thickness of the cell.

**Fig 7:**
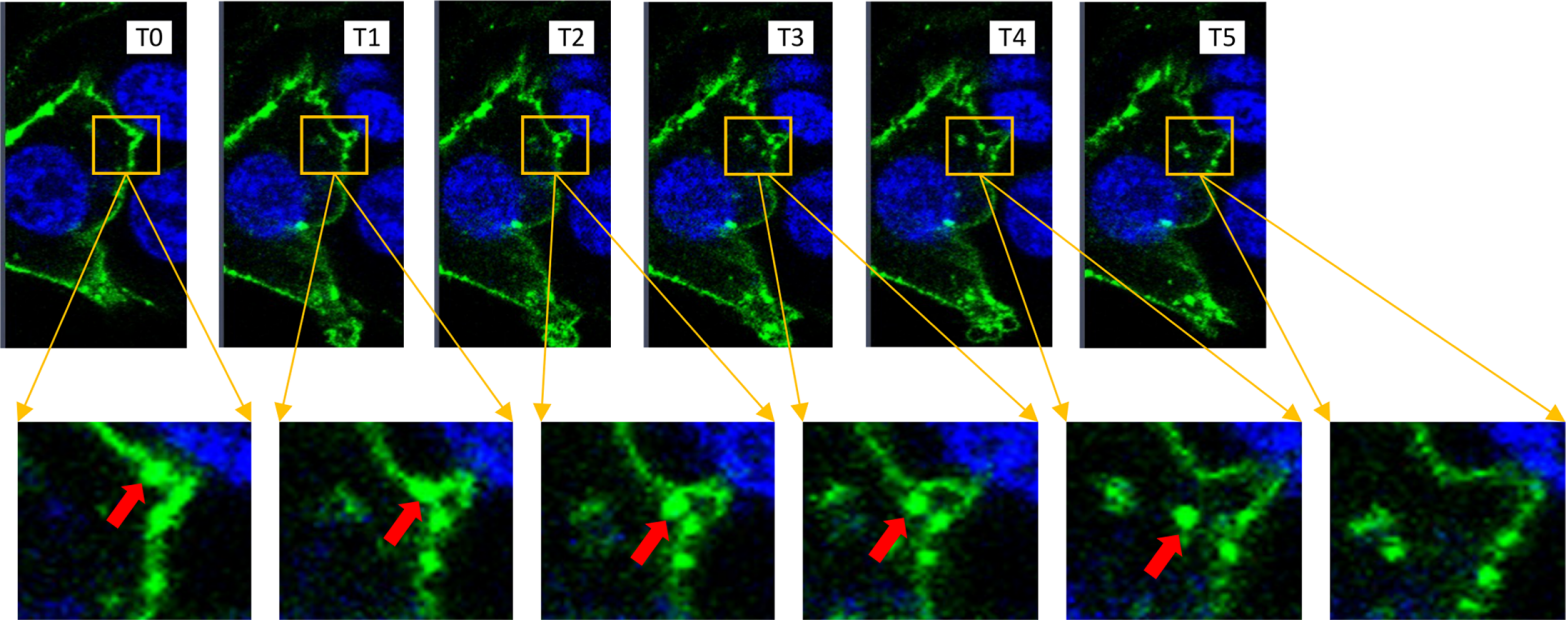
Real-time analysis showing internalization of FITC-rhGal-7. **Time-lapse imaging of FITC-rhGal-7 endocytosis in** MDA-MB-231 cells. T0: 0 sec, T1 to T5: 210 at 270 sec (at 15 seconds intervals).

To further study the entry of extracellular gal-7 inside the cells, we tagged recombinant gal-7 with FITC, allowing to follow the entry of gal-7 inside the cells using anti-FITC antibodies (**Fig 4A**). Western blot analysis of cytosolic and mitochondrial extracts of MDA-MB-231 cells using anti-FITC antibodies confirmed the entry of extracellular FITC-tagged gal-7 in the cytosol and mitochondria (**Fig 4B**). The entry of extracellular FITC-labeled gal-7 inside MDA-MB-231 cells was confirmed by confocal microscopy analysis (**Figs 5 and 6**). Successive imaging using live confocal microscopy revealed that entry of gal-7 occurred within minutes following a multistep process initiated by the binding and aggregation of FITC-gal-7 at the membrane level followed by a progressive accumulation in the cytosol (**Fig 7**).

## Discussion

Our results suggest that extracellular gal-7 controls the intracellular pool of gal-7. It does so via two distinct yet complementary mechanisms: firstly by increasing the transcriptional activation of *lgals7* gene transcription, and secondly via re-entry into the cells. These findings are of great importance in the design of gal-7 inhibitors for the treatment of various diseases where gal-7 plays a central role, most notably in cancer [6,7,9]. These findings suggest that targeting extracellular gal-7 using either CRD-specific inhibitors [14] or dimer-disrupting peptides (DIPs) [13] may be more efficient than expected for targeting intracellular gal-7-mediated interactions. Furthermore, our results showing that extracellular gal-7 induces *de novo lgals7* gene activation and our approach using FITC-tagged recombinant gal-7 to follow the fate of extracellular gal-7 inside the cells provide new and original *in vitro* model systems to investigate the inhibitory activity of these gal-7-specific inhibitors. They also provide a simple and reliable experimental platform to identify and study membrane receptors that bind extracellular gal-7.

Historically, galectins have been mostly known for their presence outside the cells following their release via a non-conventional pathway and their entry into the cells into endosomal compartments. This paradigm is based on solid experimental evidence obtained in studies that focused largely on gal-1 and gal-3. For example, Lepur et al., have shown that gal-3 enter macrophage-like cells via early endosomes rapidly (within 5–10 min) and to non-degradative vesicles, where it remains detectable for at least 24 h [11]. Such rapid re-entry of gal-3 inside cells has also been shown in cells of epithelial origin [15]. These findings with gal-1 and -3, together with our results with gal-7, support the idea that entry of extracellular galectins inside cells is a common mechanism adopted by multiple members of the galectin family. The level of redundancy shared by the different members of the galectin family with regards to the identity of glycoreceptors involved and the intracellular trafficking pathways that mediate such re-entry into cells remains, however, to be established. Although a detailed mechanism underlying entry of gal-7 is beyond the scope of this study, our preliminary results with Dynasore and Pitstop-2, two small molecular weight inhibitors of endocytosis, suggest that gal-7 enter cells via clathrin-mediated endocytosis, a major pathway for internalization of cell surface glycoproteins in mammalian cells (**S3 Fig**) [16]. Dynasore inhibits endocytic pathways by rapidly blocking coated vesicle formation via its interaction with dynamin [17] while Pitstop-2 is well known for its ability to inhibit clathrin-mediated endocytosis [18]. Clathrin-coated endocytosis has been shown to be used by other galectins, including gal-1 and gal-3, for their entry into cells [11, 19]. The inhbition observed with these inhibitors was, however, only partial for both MDA-MB-231 and OVCAR cells, suggesting that gal-7 may also enter cells via a clathrin-independent pathway as well. This would be consistent with the lack of inhibition observed with CPZ, another inhibitor normally associated with clathrin-mediated endocytosis (**S3 Fig**). The entry of gal-7 may thus depend on the cell type and/or the receptors involved. This has been well documented for gal-3 [11]. One must, however, be careful in the interpretation of experimental results obtained from using such pharmacological inhibitors. Many inhibitors of endocytosis have cell-specific effects [20, 21]. Notwithstanding these limitations, interesting questions arise: what happens in tissue expressing more than one extracellular galectin? Do they compete with each other for the same receptors/endocytic pathways? Do they have overlapping or competitive functions? This is a critical issue if one wants to inhibit the protumoral functions of galectins. We and others have already provided evidence that multiple galectins are expressed simultaneously in different tissues, most notably in prostate and breast cancer tissues [22–24].

The functions of endocytosed galectin-7 inside the cells remain unclear at present. Previous studies have shown that gal-7 can bind bcl-2 and translocates to mitochondria to control apoptosis [6,25]. Interestingly, our preliminary results suggest that endocytosed gal-7 is found in mitochondrial protein extracts. Additional, however, experiments will be necessary to confirm the presence of gal-7 in mitochondria and whether endocytosed en gal-7 gains access to mitochondrial interactors such as bcl-2. Although this is not a common pathway, exogenous proteins or endosomal proteins have been shown to reach mitochondria after leaving the endo-lysosomal compartments [26, 27]. The ability of gal-1 to re-enter breast cancer cells and to translocate to the nucleus has been recently shown to control cell invasiveness [28]. Given the fact that galectins can be released by stromal cells, these results suggest that cancer cells may take advantage of extracellular galectins that are released by neighboring cells to ensure their own survival. This is a real possibility supported by previous findings showing that gal-7 can be expressed by stromal cells, including infiltrating T cells [29]. Such mechanism would be reminiscent of HMGB1, a protein that is normally located inside the cells but that is released via a non-classical mechanism in danger situation [30,31]. Following its re-entry into cells, cytosolic HMGB1 triggers programmed cell death, possibly by interfering with beclin1-Bcl-2 complexes [32,33]. Interestingly, galectins show a number of structural and functional similarities with HMGB1. In fact, galectins are increasingly recognized as alarmins, just like HMGB1 [34–36]. Whether re-entry is dependent of the glycan-binding site of gal-7 or whether other galectins that share fine specificities with gal-7 is currently unknown. These issues are currently being investigated.

## Supporting information

S1 Fig Activation of galectin-7 promoter by human recombinant galectin-7 in MCF-7 cells. Luciferase activity measured in protein extracts collected from MCF-7 cells transfected with a luciferase reporter vector containing *p200-gal7* promoter following treatment with rhGal-7. Statistical analysis were carried out using Student’s t test for unpaired samples (** *p* ≤ 0.001).
(TIFF)

S2 Fig Effect of cycloheximide of intracellular pool of gal-7 in MDA-MB-231 cells. Cells were treated 4 h with cycloheximide (CHX; 20 μM) before addition of rhGal-7 for 16h. Cells were then harvested and intracellular gal-7 protein levels measured by Western blot using anti-gal-7 antibodies. The results are representative of two independent experiments.
(TIFF)

S3 Fig Inhibition of internalization of galectin-7. (**A**) Western blot analysis showing expression of intracellular galectin-7 in MDA-MB-231 cells after treatment (15 min) with rhGal-7 (5 μM) in absence or presence of Pitstop-2 (30 μM). (**B**) Expression of cytosolic or mitochondrial gal-7 in absence or presence of Pitstop-2 and rhGal-7 (5 μM) following a 15 min treatment of MDA-MB-231 cells. Tubulin and CoxIV were used as controls for cytosolic and mitochondrial extracts. (**C**) Effect of chlorpromazine (CPZ; 25 μM) and anrtimycin A (AMA; 1 μM) on galectin-7 expression in MDA-MB-231 cells. Cells were treated 3 h with the inhibitors before addition of rhGal-7 for 16h. (**D**) Western blot analysis of cell lysates from OVCAR-3 cells showing intracellular galectin-7 following a 15 min treatment with rhGal-7 (5 μM) in absence or presence of Pitstop-2 (30 μM) or Dynasore (30 μM). Actin was used as a control for A, C, and D experiments. Data are representative of three independent experiments.
(TIFF)
