# Supplementary figures and images for: Intracellular galectin-7 expression in cancer cells results from an autocrine transcriptional mechanism and endocytosis of extracellular galectin-7
Source: PLoS One. 2017 Nov 8;12(11):e0187194. doi: 10.1371/journal.pone.0187194 (PMC5678874; doi:10.1371/journal.pone.0187194)

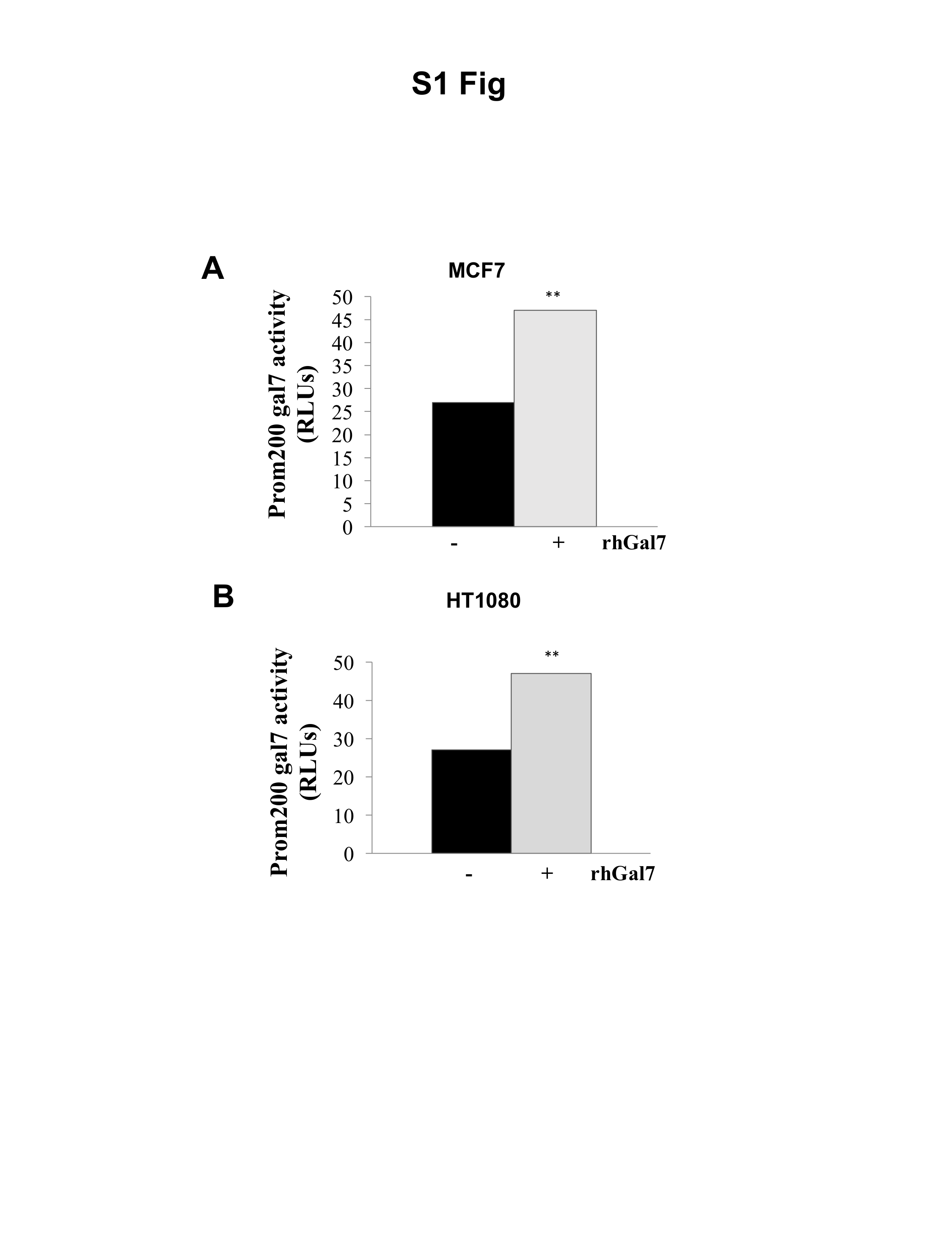

Supplement: S1 Fig — Luciferase activity measured in protein extracts collected from MCF-7 cells transfected with a luciferase reporter vector containing p200-gal7 promoter following treatment with rhGal-7. Statistical analysis were carried out using Student’s t test for unpaired samples (** p ≤ 0.001). (TIFF) [file pone.0187194.s001.tiff]

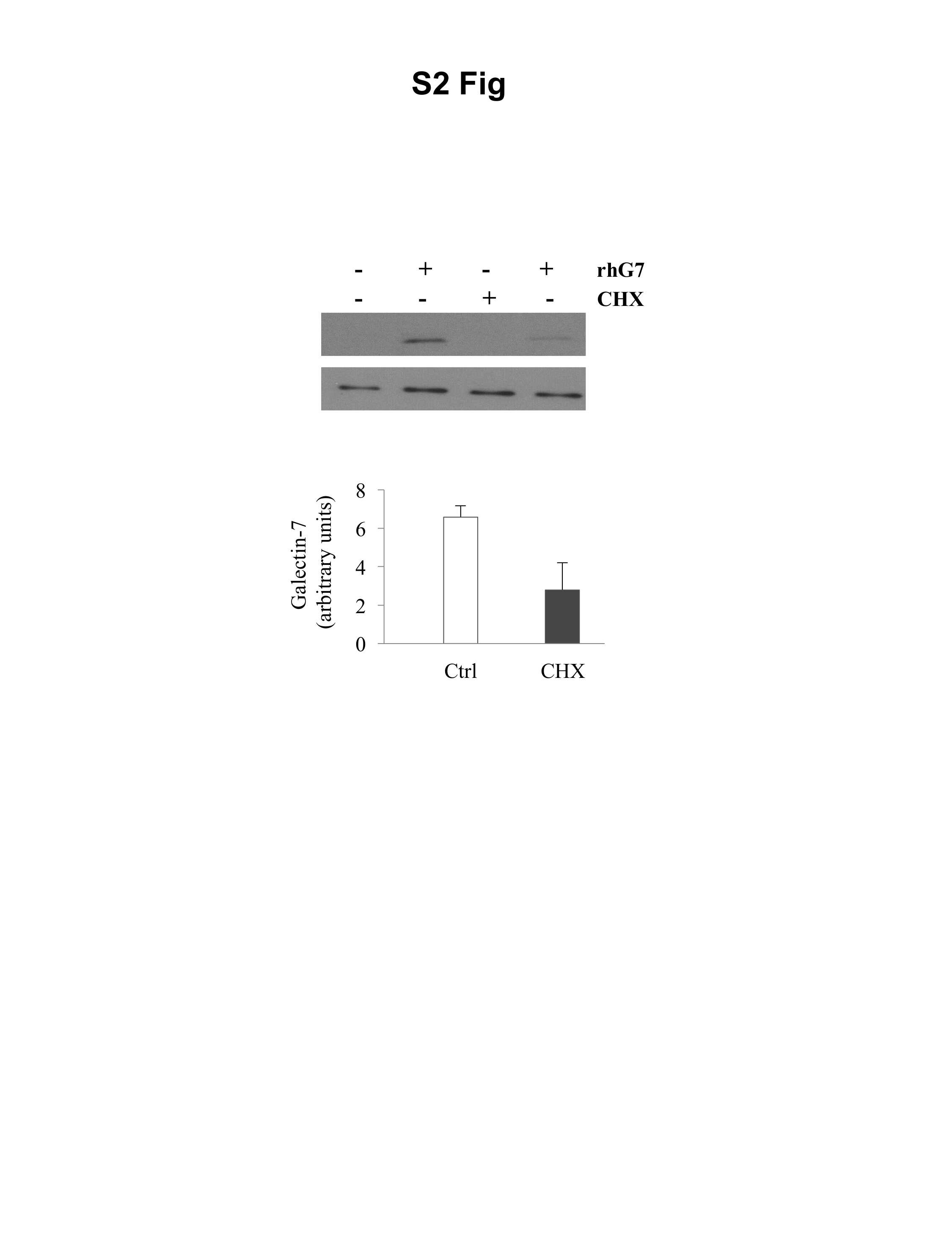

Supplement: S2 Fig — Cells were treated 4 h with cycloheximide (CHX; 20 μM) before addition of rhGal-7 for 16h. Cells were then harvested and intracellular gal-7 protein levels measured by Western blot using anti-gal-7 antibodies. The results are representative of two independent experiments. (TIFF) [file pone.0187194.s002.tiff]

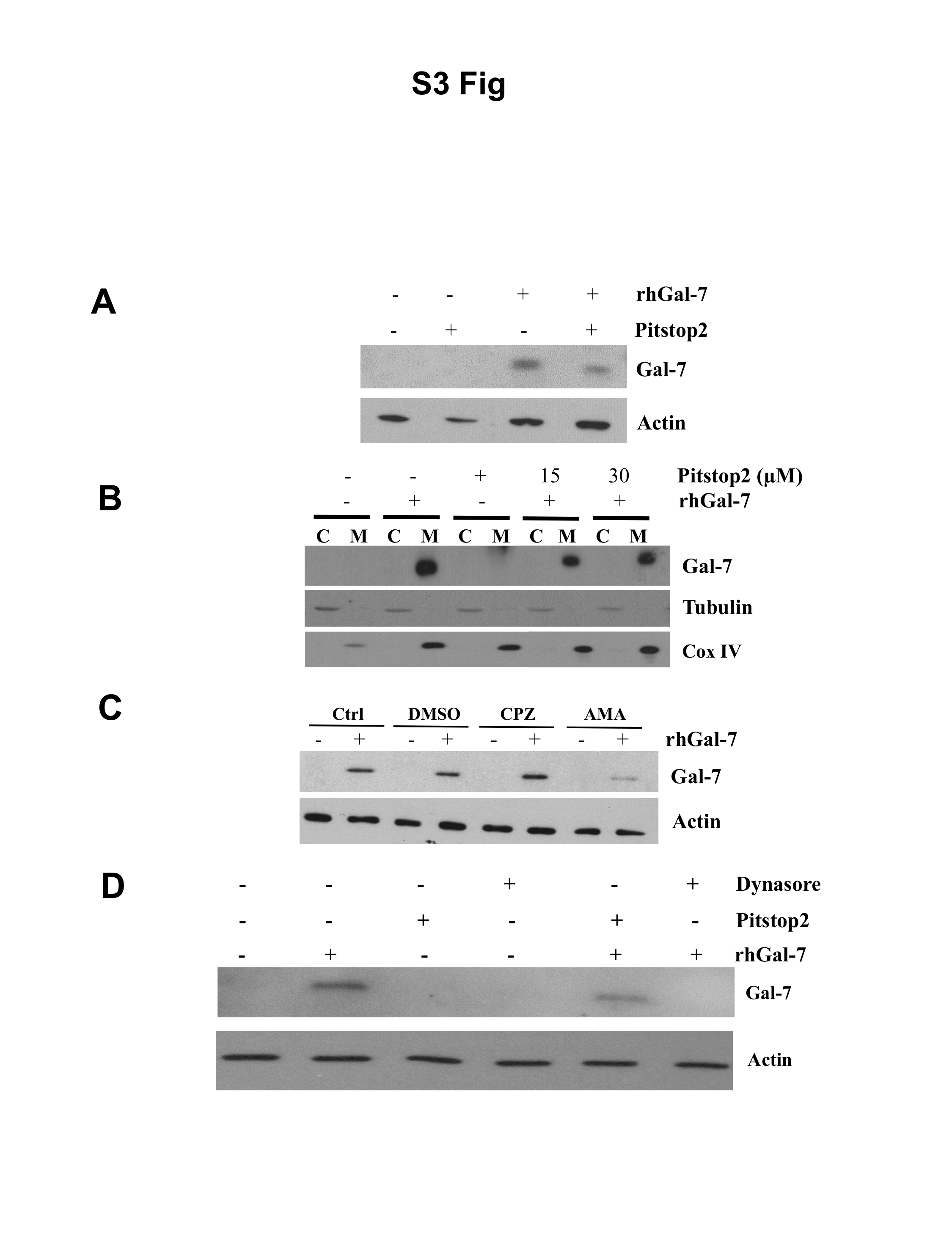

Supplement: S3 Fig — (A) Western blot analysis showing expression of intracellular galectin-7 in MDA-MB-231 cells after treatment (15 min) with rhGal-7 (5 μM) in absence or presence of Pitstop-2 (30 μM). (B) Expression of cytosolic or mitochondrial gal-7 in absence or presence of Pitstop-2 and rhGal-7 (5 μM) following a 15 min treatment of MDA-MB-231 cells. Tubulin and CoxIV were used as controls for cytosolic and mitochondrial extracts. (C) Effect of chlorpromazine (CPZ; 25 μM) and anrtimycin A (AMA; 1 μM) on galectin-7 expression in MDA-MB-231 cells. Cells were treated 3 h with the inhibitors before addition of rhGal-7 for 16h. (D) Western blot analysis of cell lysates from OVCAR-3 cells showing intracellular galectin-7 following a 15 min treatment with rhGal-7 (5 μM) in absence or presence of Pitstop-2 (30 μM) or Dynasore (30 μM). Actin was used as a control for A, C, and D experiments. Data are representative of three independent experiments. (TIFF) [file pone.0187194.s003.tiff]
